# Neonatal Outcomes of Mothers with Syphilis During Pregnancy: A Retrospective Single Center Experience

**DOI:** 10.3390/children12030307

**Published:** 2025-02-28

**Authors:** Danilo Buonsenso, Francesca Raffaelli, Anna Camporesi, Barbara Fiori, Rosalba Ricci, Lucio Romano, Marco De Santis, Giovanni Vento, Carlo Torti, Enrica Tamburrini, Piero Valentini

**Affiliations:** 1 Department of Woman and Child Health and Public Health, Fondazione Policlinico Universitario Agostino Gemelli IRCCS, 00136 Rome, Italy; marco.desantis@policlinicogemelli.it (M.D.S.); giovanni.vento@unicatt.it (G.V.); piero.valentini@unicatt.it (P.V.); 2 Area Pediatrica, Dipartimento di Scienza Della Vita e Sanità Pubblica, Università Cattolica del Sacro Cuore, 00136 Rome, Italy; 3 Dipartimento di Scienze Mediche e Chirurgiche, UOC di Malattie Infettive, Fondazione Policlinico Universitario Agostino Gemelli IRCCS, 00136 Rome, Italy; francesca.raffaelli@policlinicogemelli.it (F.R.); carlo.torti@unicatt.it (C.T.); enrica.tamburrini@unicatt.it (E.T.); 4 Anesthesia and Intensive Care Unit, “Vittore Buzzi” Children’s Hospital, 20154 Milan, Italy; anna.camporesi@asst-fbf-sacco.it; 5 Dipartimento di Scienze di Laboratorio e Infettivologiche, Fondazione Policlinico Universitario Agostino Gemelli IRCCS, 00136 Rome, Italy; barbara.fiori@policlinicogemelli.it (B.F.); rosalba.ricci@policlinicogemelli.it (R.R.); lucio.romano@policlinicogemelli.it (L.R.); 6 Dipartimento Sicurezza e Bioetica, Sezione Malattie Infettive, Università Cattolica del Sacro Cuore, 00136 Rome, Italy

**Keywords:** syphilis, congenital syphilis, pregnancy

## Abstract

Background/Objectives: Syphilis during pregnancy can be easily missed with potential severe outcomes of the newborns, including congenital syphilis (CS). We report the neonatal outcomes of a cohort of mothers with syphilis during pregnancy. Methods: a retrospective cohort study in a referral university hospital in Rome, enrolling mother/newborn couples followed up from 2016 to 2023 by a multidisciplinary team including infectious disease specialists, obstetricians, microbiologists, neonatologists and pediatricians. Primary outcome was the assessment of risk factors for development of congenital syphilis (CS) in the newborns. Results: Fifty-three pregnant women (median age 34, IQR 29–37 years) with documented syphilis in pregnancy have been included in this study. 50/52 (96.2%) were treated during pregnancy, and forty of them (80%) received adequate treatment. Fifty-three newborns were born from mothers with syphilis during pregnancy (female 25/48, 52.1%). Four newborns were classified as CS (7.5%), and two newborns as probable CS (3.8%). Newborns with CS were born more frequently from mothers treated inadequately (*p* 0.02), had higher probability of neonatal intensive care unit admission (*p* < 0.001), had a higher Venereal Disease Research Laboratory (VDRL) titer (*p* 0.076), higher ALT (*p* 0.005). Univariate logistic regression conducted on the development of CS showed an adequate treatment as a protective factor (OR 0.03; 95% CI: 0.002; 0.31; *p* = 0.002), while later weeks of pregnancy for the beginning of treatment as a risk factor (OR 1.24; 95% CI: 1.02; 1.51; *p* = 0.026). Conclusions: Syphilis still represents a potential problem for women of childbearing age and their newborns, even in a high-income setting, making congenital syphilis far from being eradicated in Italy. Moreover, adequate and early treatment should be provided to avoid negative consequences to the newborns.

## 1. Introduction

Since the COVID-19 pandemic, there has been a significant resurgence of sexually transmitted infections (STIs), particularly in young adults, including women of childbearing age, even in high-income countries [1,2,3,4,5,6,7]. Among the increase in STIs, a significant increase of syphilis cases has been reported [8]. Although syphilis can be a potentially severe infection in any age group, nowadays with effective treatments young adults are usually successfully managed. Conversely, when a pregnant woman is affected, particularly if initially misrecognized, the infection can lead to several complications. Syphilis during pregnancy can lead to miscarriage, perinatal death, prematurity, intrauterine growth retardation, or to vertical transmission leading to congenital syphilis (CS) [9,10,11,12].

Although most children with CS are asymptomatic at birth, if the infection is not diagnosed and treated, they can develop signs and symptoms later during life [13] leading to permanent sequelae [14]. Since CS is potentially preventable and treatable, most countries introduced a universal prenatal screening to treat pregnant women with syphilis and prevent the development of CS. Penicillin is the treatment of choice for syphilis in pregnancy. Treatment is considered effective if a pregnant woman is treated with three weekly administrations with intramuscular penicillin at a dose of 2,400,000 IU each (total dose, 7,200,000 IU), to be completed at least thirty days before delivery [10].

Although the World Health Organization (WHO) launched a global effort for the global eradication of CS in 2007 [15], establishing a target rate under 0.5 cases per 100,000 live births, national CS data showed significant differences among countries or even different areas within the same country [16]. For example, confirmed cases and rates of congenital syphilis by country and year varies from 14 cases in Bulgaria (rate 21.5 per 100,000) to 2 in Spain (rate 0.5 per 100,000), to no confirmed cases in several western Europe countries, although several ones have missing notifications, suggesting a possible under-recognition or under-notification, including Italy [16].

For these reasons, we believe that it is plausible to get more data on the outcomes of syphilis in pregnant women and the risk of CS in their newborns. Therefore, we performed this study aiming to better understand factors associated with CS in newborns born from mothers with syphilis during pregnancy, to plan future interventions and better prevention campaigns.

## 2. Materials and Methods

We performed a single-center, retrospective cohort study conducted at the pediatric infectious disease and adult infectious disease units of “A. Gemelli” University Hospital in Rome, Italy. We enrolled pregnant women diagnosed with syphilis between January 2016 to December 2023 and their newborns. In our hospital, a specialized team of infectious diseases take care of the maternal infection during pregnancy, along with obstetrician which are responsible for the monitoring of pregnancy, including obstetric ultrasound and delivery. After delivery, newborns are either admitted if clinically symptomatic and suspected to have CS, if asymptomatic they are referred to a long-term follow up coordinated by Pediatric Infectious Diseases specialists. The study was approved by the Ethic committee of Fondazione Policlinico Universitario A. Gemelli IRCCS, Rome (Prot 0001673, 9 October 2024).

Maternal infection was defined according to national and international guidelines [15]. The following information were collected: demographics, such as age, country of origin, HIV co-infection, outcome of the pregnancy, Venereal Disease Research Laboratory (VDRL), *T. pallidum* hemagglutination test (TPHA), time of diagnosis of syphilis, stage of disease in pregnancy, maternal therapy with dosages and times, adequacy of treatment. Mothers were defined as having received an adequate treatment if they received a penicillin regimen according to their stage of disease, if treatment was administered more than 4 weeks before delivery, and if documented test results showed evidence of a fourfold decrease of non-treponemal titer.

### 2.1. Laboratory Evaluation

For each woman and live newborn, we collected information about the following serum tests, as per routine clinical practice:

- VDRL test
- TPHA test

In case the newborn was suspected to have CS, we collected the following data:

- Complete blood count
- Liver function
- Cerebrospinal fluid (CSF) examination, including CSF white blood cell count if >25/mL, CSF protein, and whether the non-treponemal tests on CSF was reactive [17,18]. Central nervous system involvement was defined as a positive VDRL on the CSF.
- Imaging: abdominal ultrasound, brain ultrasound or magnetic resonance imaging, full body X-ray. Cranial ultrasonography (CUS) was performed by experienced neonatologists, as for routine clinical care.
- Fundus oculi examination
- Clinical signs and symptoms, including skin rash, hepatosplenomegaly, cholestatic jaundice.

A newborn was eventually diagnosed with CS if one or more of the following criteria were satisfied:

- Symptoms suggestive of CS;
- Titer of the neonatal non-treponemal test was fourfold higher than the maternal test at the time of delivery;
- Persistent positivity of serology after 12 months of life.

In all the other cases, newborns were only considered exposed in utero to *T. pallidum* but not infected.

According to local practice and international guidelines [15], infants diagnosed with CS were treated with either intravenous aqueous penicillin G or with a single dose of intramuscular benzathine penicillin according to a case-by-base decision of the assessing physician.

In addition, according to local practice and international guidelines [15], independently from the neonatal diagnosis, newborns born from women with syphilis were followed up every three to six months for up to 18 months for the purpose of this study.

### 2.2. Statistical Analyses

This is an observational analysis without any specific a priori hypothesis. We enrolled all patients who came to our attention during the study period with the precise aim to describe a disease and its outcomes which are often neglected and under-attentioned by the medical community in the optimistic belief that it is not a sanitary problem in a first-world country in 2024. Results were expressed as N (%) for categorical measures and mean ± SD or median (IQR) as appropriate in case of continuous variables. Associations between variables of interest were studied with Pearson’s Chi squared test or Fisher’s exact test as appropriate in case of categorical variables and with Student’s T test or Wilcoxon Rank-Sum test as appropriate in case of continuous variables. Regression models were applied to study the effect of clinically pertinent covariates on continuous outcomes and logistic regression models were applied on dichotomous outcomes. Changes in antibody levels over time were studied with mixed-effects models for correlate outcomes, incorporating random effects to account for individual-level variation. Data were analyzed with Stata 18.0 B.E. (StataCorp LLC, College Station, TX, USA). All statistical tests were two-sided and the level of statistical significance was set at 0.05.

## 3. Results

### 3.1. Maternal Data

Fifty-three HIV-negative pregnant women (median age 34, IQR 29–37 years) with documented syphilis in pregnancy have been included in this study. Most women were of non-Italian origin. Two women had a secondary syphilis with skin rash. The remaining fifty-one women had no symptoms and had a latent stage of syphilis. Further details about the maternal population are reported in Table 1.

Among 53 pregnant women, 50/52 (96.2%, one missing data) were treated during pregnancy, forty of them (80%) receiving an adequate treatment. The women who were not treated during pregnancy were diagnosed after delivery. TPHA and VDRL levels were analyzed with multilevel mixed linear regression for correlated outcomes to assess the effect of an adequate treatment on them. TPHA levels did not significantly change from baseline to first follow-up nor to second follow up, independently from the mothers having received adequate or inadequate treatment (*p* 0.89). Similarly, VDRL levels showed a reduction after treatment which was however not significant.

### 3.2. Neonatal Data and Outcomes

Fifty-three newborns were born from mothers with syphilis in pregnancy (female 25/48, 52%). Four newborns were classified as CS (7.5%), and two newborns as probable CS (3.7%). Two newborns (3.7%) were classified as small for gestational age (SGA). Three newborns required admission to the neonatal intensive care unit. Other characteristics of the neonatal population are reported in Table 2.

There was a linear correlation between baseline maternal TPHA and neonatal TPHA at birth and the follow-up maternal TPHA and neonatal TPHA. Conversely, VDRL levels in the newborns were not associated with VDRL levels in mothers. We analyzed if the route of administration of therapy to babies (intravenous or intramuscular) influenced the levels of TPHA and VDRL in the follow-ups, but it was not significant.

Table 3 shows the main characteristics of newborn with or without a diagnosis of CS. Newborns with CS were more frequently born from mothers treated inadequately (*p* 0.02), had higher probability of NICU admission (*p* < 0.001), had a higher VDRL titer (*p* 0.076), a higher probability of receiving a lumbar puncture (0.008), higher AST (*p* 0.089) and ALT (*p* 0.005).

Table 4 shows the main characteristics of newborn with a diagnosis of probable or confirmed CS versus those without a diagnosis of CS. Newborns with CS were more frequently born from mothers treated inadequately (*p* < 0.001), had higher probability of NICU admission (*p* 0.005), had a higher VDRL titer (*p* 0.076), a higher probability of receiving a lumbar puncture (*p* < 0.001), had higher ALT (*p* 0.009). 

Table 5 shows characteristics of newborns according to adequacy of maternal treatment. Newborns from mothers with inadequate treatment had higher probability of NICU admission (*p* < 0.061), close to statistical significance, and to receive a lumbar puncture (*p* < 0.001) and had higher ALT levels (*p* 0.039).

### 3.3. Regression Models

Univariate logistic regression conducted on outcome “congenital syphilis” showed a protective role of adequate treatment (OR 0.07; 95% CI: 0.007–0.82; *p* = 0.034).

**Figure 1:**
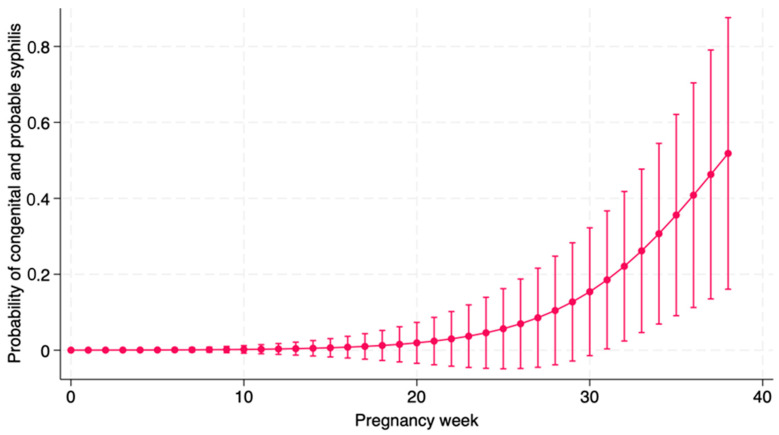
Probability of having Congenital Syphilis according to the week of beginning treatment in pregnancy.

Univariate logistic regression conducted on the outcome “congenital + probable syphilis” showed a protective effect of adequate treatment (OR 0.03; 95% CI: 0.002; 0.31; *p* = 0.002) and a risk factor for week of pregnancy of start of treatment (OR 1.24; 95% CI: 1.02; 1.51; *p* = 0.026), as showed in Figure 1.

Details of the cases of probable and confirmed CS are reported in Table 6.

## 4. Discussion

In this paper, we found that despite WHO efforts to eradicate CS throughout universal maternal screening, in the last six years we had seven cases of CS (four of them confirmed, two as probable), highlighting that even in high income countries, access to antenatal screening to eradicate syphilis has gaps. Congenital syphilis, in fact, is theoretically easily preventable. However, syphilis cases are frequently missed during pregnancy, leading to missed opportunities to start adequate treatment in the affected women. In fact, the most severe cases of CS were all diagnosed in newborns whose mothers were not screened in pregnancy and recognized to have syphilis only after the diagnosis of CS was done in newborns.

More than half of the women diagnosed with syphilis in pregnancy in our center were non-Italian, mostly from Eastern Europe and Latin America, in line with an old report performed in multiple Italian centers [19]. In line with a recent paper published by colleagues from Naples, Southern Italy, among foreign mothers there was a prevalence of women from Eastern Europe [20]. This is a critical point as in these countries there are no active policies nor monitoring plans of CS [16], making women from these areas probably less aware of the importance of being screened for syphilis during pregnancy. Of note, awareness programs should not be focused to women only. In our cohort, in fact, about 82% of partners were eventually found to be positive to syphilis tests as well. This highlights the importance of performing syphilis screening more than once in pregnant women accessing antenatal care, particularly if risk factors such as multiple partners are noted.

As expected, we found that adequacy of the maternal treatment is the most important factor to prevent CS in the newborn. Maternal treatment is considered effective if a pregnant woman is treated with three weekly administrations with intramuscular penicillin at a dose of 2,400,000 IU each (total dose, 7,200,000 UI), to be completed at least thirty days before delivery [10]. In fact, also the latest the diagnosis of syphilis was performed during pregnancy, the higher the risk for the newborn to develop CS, probably because a late diagnosis does not allow to complete treatment on time. Our data, demonstrated in Figure 1, clearly show a correlation between delayed treatment during pregnancy and higher risk of confirmed or probable CS. Consequently, our data further reinforce the importance of testing mothers not only at the beginning of pregnancy and close to delivery, but even months earlier, to have enough time to complete the treatment.

About the cases of CS, the clinical and laboratory findings of our cohort were in line with what expected. Children presented the typical symptoms including palmoplantar rash, higher VDRL titers, and blood abnormalities including lower platelet counts and higher liver enzymes, specifically AST, in line with what expected from literature [21,22]. Of note, all our cases were treated successfully using intravenous penicillin G or, in probable cases, with benzathine penicillin G (50,000 U/kg intramuscularly as a single dose). We did not have deaths in children with CS, probably because all cases were diagnosed within the first two months of life.

The main limitation of our study is the retrospective nature and the low number of children with confirmed or probable CS, somehow limiting the statistical power of some comparisons, which does not allow for post-hoc analyses, but it is to be intended as a sign of alarm and a call for colleagues no to lose vigilance on an oten under-attentioned disease which can have deleterious consequences. It is also representative of the local epidemiology only. Therefore, a multicenter nationwide study will be the next step to provide further information on a national basis. In addition, we have not collected data about other sexually transmitted infections other than HIV, as all mothers were HIV negative. On the other side, the extensive characterization of the cohort and advanced statistical studies are strengths of our manuscript, dealing with a relatively neglected conditions that is significantly raising across the globe.

## 5. Conclusions

In conclusion, we documented that syphilis is still a problem among women of childbearing age and their newborns, even in a high income setting like Rome, making CS far from being eradicated in Italy, despite WHO reports reported no cases in Italy since several years [16]. More interventions are needed to guarantee access to syphilis screening and adequate treatments to all women in pregnancy, hopefully through multiple assessments, to prevent a serious, but preventable complications, as CS is.

## Figures and Tables

**Table 1:** Characteristics of mothers with syphilis during pregnancy. Data are presented as N (%) or median (IQR).

		Total
		N = 53
Age, years		34.0 (29.0–37.0)
Country	Italy	17 (32.1%)
	Romania	16 (30.2%)
	Peru	3 (5.7%)
	India	1 (1.9%)
	Moldavia	5 (9.4%)
	Egypt	1 (1.9%)
	Poland	2 (3.8%)
	Ukraine	2 (3.8%)
	Morocco	1 (1.9%)
	Nigeria	1 (1.9%)
	Russia	1 (1.9%)
	Ecuador	1 (1.9%)
	Albania	1 (1.9%)
	Brazil	1 (1.9%)
Drug user		1 (1.9%)
Previous pregnancies		26/51 (51.0%)
Previous syphilis		10/53 (18.9%)
Previous treatment		8/53 (15.1%)
VDRL positivity		33/51 (64.7%)
VDRL titer		8.0 (4.0–32.0)
TPHA titer		2560.0 (1280.0–5120.0)
Symptoms		3/53 (5.7%)
Syphilis Therapy during pregnancy		50/52 (96.2%)
Compliance to treatment		50/52 (96.2%)
Adequate treatment		40/52 (76.9%)
Follow Up (FU) time, days		96.0 (55.0–145.0)
VDRL at FU#1 positivity		18/39 (46.2%)
VDRL at FU#1 titer		1/8.0 (1/4.0–1/32.0)
TPHA at FU#1		1/1280 (1/640–1/5120)

TPHA: Treponema Pallidum Haemagglutination Assay. VDRL: Venereal Disease Research Laboratory, FU: follow-up.

**Table 2:** Characteristics of newborns from women with syphilis during pregnancy.

		Total
		N = 53
Sex	Male	23 (43.4%)
	Female	25 (47.2%)
	Missing	5 (9.4%)
Dead fetus		1/53 (1.9%)
Prenatal US		38/42 (90.5%)
Gestational age (days)		273.5 (262.0–281.0)
Weight of fetus (g)		3210.0 (2910.0–3620.0)
Small for Gestational Age		4/43 (9.3%)
Weight Z score		0.1 (−0.7–0.7)
Weight Percentile		56.0 (21.0–77.0)
Head circumference		34.0 (33.0–35.0)
Head circumference Z score		−1.3 (−2.3–0.3)
Head circumference percentile		71.0 (32.0–83.0)
Neonatal Intensive Care Unit admission		3/31 (9.7%)
Birth-to-serology distance, days		0.0 (0.0–2.0)
Time serology baby		1.0 (1.0–1.5)
VDRL baby positivity		12/43 (27.9%)
VDRL baby titer		8.0 (8.0–32.0)
TPHA baby		2560.0 (1280.0–5120.0)
Lumbar puncture done		9/53 (17%)
CSF Protein pathologic		4/9 (44.4%)
CSF Vdrl positivity		3/8 (37.5%)
Hemoglobin, gr/dL		17.8 (14.4–19.9)
Platelets, N × 10^9^/L		228.0 (159.0–283.0)
Asparatate aminotransferase, U/L		38.0 (0.0–65.0)
Alanin aminotransferase, U/L		13.0 (8.0–21.0)
Brain US positivity		4/15 (26.7%)
Abdominal US positivity		2/17 (11.8%)
CS treatment		6/43 (14.0%)
Baby FU1 distance (days)		49.0 (12.0–206.0)
Baby FU1 Vdrl positive		2/19 (10.5%)
Baby FU1 TPHA titer		640.0 (160.0–1280.0)
Congenital syphilis		4/53 (7.5%)
Probable syphilis		2/53 (3.8%)

VDRL: Venereal Disease Research Laboratory, TPHA: *T. pallidum* hemagglutination test, CFS: cerebrospinal fluid, US: ultrasound, CS: Congenital Syphilis, FU: follow-up.

**Table 3:** Comparison between newborns with or without congenital syphilis (CS).

		Total	No CS	CS	*p*-Value
		N = 53	N = 49	N = 4	
Maternal Age		34.0 (29.0–37.0)	34.0 (29.0–37.0)	33.0 (25.5–40.0)	0.89
Vdrl titer in mother		8.0 (4.0–32.0)	8.0 (4.0–32.0)	16.0 (16.0–16.0)	0.6
Drug-user		1 (1.9%)	1 (2.0%)	0 (0.0%)	0.77
Co-infection		1 (1.9%)	1 (2.0%)	0 (0.0%)	0.77
Previous syphilis		10 (18.9%)	8 (16.3%)	2 (50.0%)	0.098
Previous treatment		8 (15.1%)	8 (16.3%)	0 (0.0%)	0.38
TPHA titer in mother		2560.0 (1280.0–5120.0)	2560.0 (1280.0–5120.0)	3200.0 (1280.0–5120.0)	0.62
Mother Therapy		50 (96.2%)	46 (95.8%)	4 (100.0%)	0.68
Adequate treatment		40 (76.9%)	39 (81.2%)	1 (25.0%)	0.01
Delivery type	Vaginal	37 (69.8%)	34 (69.4%)	3 (75.0%)	0.94
	Cesarean	15 (28.3%)	14 (28.6%)	1 (25.0%)	
	Missing	1 (1.9%)	1 (2.0%)	0 (0.0%)	
Sex	Male	23 (43.4%)	22 (44.9%)	1 (25.0%)	0.48
	Female	25 (47.2%)	22 (44.9%)	3 (75.0%)	
	Missing	5 (9.4%)	5 (10.2%)	0 (0.0%)	
Death of fetus		1 (1.9%)	1 (2.0%)	0 (0.0%)	0.77
Prenatal US		38 (90.5%)	37 (92.5%)	1 (50.0%)	0.046
Gestational age, days		273.5 (262.0–281.0)	274.5 (267.0–281.0)	265.0 (227.5–271.0)	0.064
Weight of fetus, g		3210.0 (2910.0–3620.0)	3210.0 (2910.0–3600.0)	3600.0 (1965.0–4250.0)	0.71
Head Circumference		34.0 (33.0–35.0)	34.0 (33.0–35.0)	37.0 (23.0–37.0)	0.33
NICU admission		3 (5.6%)	1 (3.4%)	2 (50.0%)	<0.001
VDRL baby positivity		12 (27.9%)	10 (25.6%)	2 (50.0%)	0.3
VDRL titer in newborn		8.0 (8.0–32.0)	8.0 (4.0–8.0)	80.0 (32.0–128.0)	0.076
TPHA baby		2560.0 (1280.0–5120.0)	2560.0 (1280.0–5120.0)	1280.0 (1280.0–3200.0)	0.55
CSF analysis performed		9 (22.5%)	6 (16.7%)	3 (75.0%)	0.008
CSF VDRL		0.0 (0.0–1024.0)	0.0 (0.0–0.0)	1024.0 (1024.0–1024.0)	0.031
Hemoglobin, g/dL		17.8 (14.4–19.9)	17.8 (14.7–20.0)	14.4 (8.7–17.8)	0.24
Platelets, N × 10^9^/L		228.0 (159.0–283.0)	255.0 (183.0–286.0)	126.5 (21.5–225.0)	0.15
Asparatate aminotransferase, U/L		38.0 (0.0–65.0)	37.0 (0.0–64.5)	232.0 (232.0–232.0)	0.089
Alanin aminotransferase, U/L		13.0 (8.0–21.0)	12.0 (7.0–17.0)	42.5 (22.0–95.0)	0.005
Brain US positivity		4 (26.7%)	2 (18.2%)	2 (50.0%)	0.22
Abdominal US positivity		2 (11.8%)	0 (0.0%)	2 (50.0%)	<0.001

VDRL: Venereal Disease Research Laboratory, TPHA: *T. pallidum* hemagglutination test, CFS: cerebrospinal fluid, US: ultrasound, CS: Congenital Syphilis, NICU: neonatal intensive care unit.

**Table 4:** Comparison between newborns with confirmed or probable congenital syphilis (CS) vs. the others.

		Total	No	Congenital + Probable	*p*-Value
		N = 53	N = 47	N = 6	
Maternal Age, years		34.0 (29.0–37.0)	34.0 (29.0–37.0)	34.0 (26.0–40.0)	0.82
VDRL titer in mother		8.0 (4.0–32.0)	8.0 (4.0–32.0)	16.0 (16.0–16.0)	0.6
Drug-user		1 (1.9%)	1 (2.1%)	0 (0.0%)	0.72
Co-infection		1 (1.9%)	1 (2.1%)	0 (0.0%)	0.72
Previous syphilis		10 (18.9%)	8 (17.0%)	2 (33.3%)	0.34
Previous treatment		8 (15.1%)	8 (17.0%)	0 (0.0%)	0.27
Positive partner		9 (16.9%)	8 (80.0%)	1 (100.0%)	0.62
Tpha titer mother		2560.0 (1280.0–5120.0)	2560.0 (640.0–5120.0)	3840.0 (1280.0–5120.0)	0.36
Therapy		50/52 (96.2%)	44 (93.6%)	6 (100.0%)	0.6
Adequate treatment		40 (76.9%)	39 (84.8%)	1 (16.7%)	<0.001
Delivery type	Vaginal	37 (69.8%)	32 (68.1%)	5 (83.3%)	0.73
	Cesarean	15 (28.3%)	14 (29.8%)	1 (16.7%)	
	Missing	1 (1.9%)	1 (2.1%)	0 (0.0%)	
Sex	Male	23 (43.4%)	22 (46.8%)	1 (16.7%)	0.16
	Female	25 (47.2%)	20 (42.6%)	5 (83.3%)	
	Missing	5 (9.4%)	5 (10.6%)	0 (0.0%)	
Death of fetus		1 (1.9%)	1 (2.1%)	0 (0.0%)	0.72
Prenatal US		38 (90.5%)	36 (94.7%)	2 (50.0%)	0.004
Gestational age, days		273.5 (262.0–281.0)	275.5 (267.5–281.0)	264.5 (244.0–271.0)	0.015
Weight of fetus, g		3210.0 (2910.0–3620.0)	3210.0 (2915.0–3600.0)	3162.5 (2520.0–4250.0)	0.94
Small for Gestational Age		4 (7.5%)	3 (6.4%)	1 (16.0%)	0.5
Weight Z score		0.1 (−0.7–0.7)	0.1 (−1.0–0.9)	0.0 (−0.7–0.7)	0.94
Weight percentile		56.0 (21.0–77.0)	56.0 (16.0–74.5)	51.0 (25.0–77.0)	0.89
Head Circumference		34.0 (33.0–35.0)	34.0 (33.0–35.0)	34.0 (32.0–37.0)	0.87
Head Circumference centile		71.0 (32.0–83.0)	71.0 (33.3–87.5)	53.0 (32.0–74.0)	0.67
NICU adm		3 (5.6%)	1 (2.1%)	2 (33.3%)	0.003
VDRL in newborn positivity		12 (22.6%)	10 (21.2%)	2 (33.3%)	0.75
VDRL titer in newborn		8.0 (8.0–32.0)	8.0 (4.0–8.0)	80.0 (32.0–128.0)	0.076
TPHA titer in newborn		2560.0 (1280.0–5120.0)	2560.0 (1280.0–5120.0)	1920.0 (1280.0–2560.0)	0.67
CSF analysis performed		9 (22.5%)	5 (14.7%)	4 (66.7%)	0.005
VDRL in CSF		0.0 (0.0–1024.0)	0.0 (0.0–0.5)	1024.0 (0.0–1024.0)	0.17
Hemoglobin, g/dL		17.8 (14.4–19.9)	17.7 (14.7–19.4)	17.8 (11.1–19.9)	0.96
Platelets, N × 10^9^/L		228.0 (159.0–283.0)	260.0 (159.0–286.0)	223.5 (28.0–225.0)	0.21
Asparatate aminotransferase, U/L		38.0 (0.0–65.0)	36.0 (0.0–65.0)	141.0 (50.0–232.0)	0.14
Alanin aminotransferase, U/L		13.0 (8.0–21.0)	11.5 (3.5–17.5)	22.0 (17.0–63.0)	0.013
Brain US positivity		4 (7.5%)	1 (2.1%)	3 (50.0%)	0.095
X ray		0 (0.0%)	0 (0.0%)	0 (0.0%)	
Abdominal us positivity		2 (3.8%)	0 (0.0%)	2 (33.3%)	0.001

VDRL: Venereal Disease Research Laboratory, TPHA: *T. pallidum* hemagglutination test, CFS: cerebrospinal fluid, US: ultrasound, CS: Congenital Syphilis, NICU: neonatal intensive care unit.

**Table 5:** Comparison of different outcomes according to maternal treatment status. Data are presented as N (%) or median (IQR).

		Total	Inadequate	Adequate	*p*-Value
		N = 52	N = 12	N = 40	
Maternal Age		34.0 (29.0–37.0)	35.0 (31.5–38.0)	33.5 (28.5–37.0)	0.29
Baseline VDRL in mother		8.0 (4.0–32.0)	16.0 (16.0–16.0)	8.0 (4.0–32.0)	0.6
Drug use		1 (1.9%)	0 (0.0%)	1 (2.5%)	0.58
Coinfection		1 (1.9%)	0 (0.0%)	1 (2.5%)	0.58
Previous syphilis		10 (19.2%)	1 (8.3%)	9 (22.5%)	0.27
Previous treatment		8 (15.4%)	0 (0.0%)	8 (20.0%)	0.092
Positive partner		9 (17.3%)	3 (25.0%)	6 (15.0%)	0.34
Baseline TPHA mother		2560.0 (1280.0–5120.0)	5120.0 (1920.0–5120.0)	1920.0 (1280.0–5120.0)	0.2
Delivery type	Vaginal	36 (69.2%)	6 (50.0%)	30 (75.0%)	0.17
	Cesarean	15 (28.8%)	6 (50.0%)	9 (22.5%)	
	Missing	1 (1.9%)	0 (0.0%)	1 (2.5%)	
Sex	Male	23 (44.2%)	5 (41.7%)	18 (45.0%)	0.36
	Female	24 (46.2%)	7 (58.3%)	17 (42.5%)	
	Missing	5 (9.6%)	0 (0.0%)	5 (12.5%)	
Congenital Syphlis		4 (7.7%)	3 (25%)	2 (2.5%)	0.01
Probable Congenital Syphlis		2 (3.8%)	2 (16.7%)	0	0.008
Death of fetus		1 (1.9%)	0 (0.0%)	1 (2.5%)	0.58
Prenatal US		37 (71.2%)	7 (58.3%)	30 (75.0%)	0.013
Gestational age (days)		274.0 (267.0–281.0)	260.5 (246.0–272.5)	278.0 (271.0–282.0)	0.003
Weight of fetus, g		3227.5 (2915.0–3620.0)	2950.0 (2255.0–3375.0)	3310.0 (2925.0–3700.0)	0.067
Small for Gestational Age		2 (3.8%)	1 (8.3%)	1 (2.5%)	0.02
Weight Z score		0.2 (−0.7–0.8)	0.5 (−0.2–1.1)	−0.0 (−1.0–0.7)	0.43
Percentile		56.0 (21.0–77.0)	62.0 (25.0–77.0)	54.0 (21.0–67.0)	0.84
Head circumference		34.0 (33.0–35.0)	33.0 (31.1–35.0)	34.0 (34.0–35.0)	0.17
NICU admission		3 (5.7%)	2 (8.3%)	1 (2.5%)	0.061
VDRL positivity in newborn		12 (28.6%)	5 (41.7%)	7 (23.3%)	0.23
VDRL titer in newborn		1/8.0 (1/8.0–1/32.0)	1/32.0 (1/4.0–1/128.0)	1/8.0 (1/8.0–1/8.0)	0.55
Baseline TPHA level in newborn		2560.0 (1280.0–5120.0)	2560.0 (1280.0–12,800.0)	2560.0 (1280.0–5120.0)	0.45
CSF analysis performed		9 (23.1%)	7 (63.6%)	2 (7.1%)	<0.001
VDRL CSF		0.0 (0.0–1/1024.0)	0.0 (0.0–0.0)	1/512.5 (1.0–1/1024.0)	0.13
Hemoglobin, g/dL		17.8 (14.7–19.9)	17.5 (13.3–17.8)	17.9 (15.2–20.2)	0.28
Platelets, N × 10^9^/L		235.0 (171.0–284.5)	226.5 (209.0–255.0)	271.5 (159.0–327.0)	0.37
Asparatate aminotransferase, U/L		37.0 (0.0–64.5)	50.0 (36.0–232.0)	34.0 (0.0–64.0)	0.21
Alanin aminotransferase, U/L		13.0 (8.0–21.0)	17.5 (12.0–27.0)	11.0 (0.0–19.0)	0.039
Brain US positivity		4 (28.6%)	2 (25.0%)	2 (33.3%)	0.73
Abdominal US positivity		2 (12.5%)	2 (40.0%)	0 (0.0%)	0.025

VDRL: Venereal Disease Research Laboratory, TPHA: *T. pallidum* hemagglutination test, CFS: cerebrospinal fluid, US: ultrasound, CS: Congenital Syphilis, NICU: neonatal intensive care unit.

**Table 6:** Main features of the newborns with probable or confirmed CS. * Probable CS.

Sex	Birth Weight (g)	GA	Maternal Origin	Prenatal Care	Maternal Treatment	CS Diagnosis: Serologic Tests and Others Investigations	CS diagnosis: Clinical Features	Long Bone X-Ray	CSF	Outcome
M	2950	37	Peru	no	Post partum	VDRL 1/32 TPHA 1/1280 Low Hb Low PLTs Hepatitis	Rash Hepatosplenomegaly	Normal	Normal	Cured (IV penicillin)
F	980	28	Romania	no	Post partum	VDRL 1/128 TPHA 1/5120 Low PLTs Hepatitis	Ascites Hepatosplenomegaly Ventricolomegaly	Normal	Normal	Cured (IV penicillin)
F	4250	38	Moldavia	yes	Non adequate	VDRL neg TPHA 1/1280	asymptomatic	Normal	VDRL reactive	Cured (IV penicillin)
F	4150	39	Moldavia	no	Non adequate	VDRL neg TPHA 1/1280	asymptomatic	Normal	VDRL reactive	Cured (IV penicillin)
F	2520	36	Romania	no	Post partum	VDRL neg TPHA 1/2560	asymptomatic	Normal	Normal	Cured (IM penicillin) *
F	3375	38	Italy	Yes	Non adequate	VDRL neg TPHA 1/2560	asymptomatic	Normal	Normal	Cured (IM penicillin) *

## Data Availability

The data presented in this study are available on request from the corresponding author. The data are not publicly available due to ethical committee restrictions.
